# Interdependency of CEACAM-1, -3, -6, and -8 induced human neutrophil adhesion to endothelial cells

**DOI:** 10.1186/1479-5876-6-78

**Published:** 2008-12-10

**Authors:** Keith M Skubitz, Amy PN Skubitz

**Affiliations:** 1 The Department of Medicine, the University of Minnesota Medical School, and the Masonic Cancer Center, Minneapolis, MN 55455, USA; 2 The Department of Laboratory Medicine and Pathology, the University of Minnesota Medical School, and the Masonic Cancer Center, Minneapolis, MN 55455, USA

## Abstract

Members of the carcinoembryonic antigen family (CEACAMs) are widely expressed, and, depending on the tissue, capable of regulating diverse functions including tumor promotion, tumor suppression, angiogenesis, and neutrophil activation. Four members of this family, CEACAM1, CEACAM8, CEACAM6, and CEACAM3 (recognized by CD66a, CD66b, CD66c, and CD66d mAbs, respectively), are expressed on human neutrophils. CD66a, CD66b, CD66c, and CD66d antibodies each increase neutrophil adhesion to human umbilical vein endothelial cell monolayers. This increase in neutrophil adhesion caused by CD66 antibodies is blocked by CD18 mAbs and is associated with upregulation of CD11/CD18 on the neutrophil surface. To examine potential interactions of CEACAMs in neutrophil signaling, the effects on neutrophil adhesion to human umbilical vein endothelial cells of a set of CD66 mAbs was tested following desensitization to stimulation by various combinations of these mAbs. Addition of a CD66 mAb in the absence of calcium results in desensitization of neutrophils to stimulation by that CD66 mAb. The current data show that desensitization of neutrophils to any two CEACAMs results in selective desensitization to those two CEACAMs, while the cells remain responsive to the other two neutrophil CEACAMs. In addition, cells desensitized to CEACAM-3, -6, and -8 were still responsive to stimulation of CEACAM1 by CD66a mAbs. In contrast, desensitization of cells to CEACAM1 and any two of the other CEACAMs left the cells unresponsive to all CD66 mAbs. Cells desensitized to any combination of CEACAMs remained responsive to the unrelated control protein CD63. Thus, while there is significant independence of the four neutrophil CEACAMs in signaling, CEACAM1 appears to play a unique role among the neutrophil CEACAMs. A model in which CEACAMs dimerize to form signaling complexes could accommodate the observations. Similar interactions may occur in other cells expressing CEACAMs.

## Background

The carcinoembryonic antigen (CEA)^2 ^family consists of two subfamilies, the CEACAM subgroup and the pregnancy specific glycoprotein (PSG) subgroup. Members of this family have been redundantly named [for review see [1-4]], but subsequent consensus unified the nomenclature for the CEACAM family [5]. CEACAM family members are widely expressed in epithelial, endothelial, and hematopoietic cells, including neutrophils, T-cells, and NK cells. CEACAMs appear to be capable of transmitting signals that result in a variety of effects depending on the tissue, including tumor suppression, tumor promotion, angiogenesis, neutrophil activation, lymphocyte activation, regulation of the cell cycle, and regulation of adhesion [2,3,5-42]. In many tissues, more than one CEACAM family member are expressed concurrently. For example, CEACAMs 1, 5, and 6 are often expressed in ovarian, endometrial, cervical, breast, lung, and colon carcinomas, and may be useful as biomarkers in cancer [43-47]. A CEACAM5 expressing measles virus has entered phase I trials in ovarian cancer [48]. CD66mAbs that recognize CEACAMs are also in clinical trials as part of conditioning regimens in allogeneic stem cell transplantation for acute leukemia [49,50]

The CEACAM gene family contains more than seventeen expressible closely related genes that belong to the immunoglobulin (Ig) gene superfamily [for review see [1,2,4,5,22] and **cea**.klinikum.uni-muenchen.de]. Each of the human CEACAM family molecules contains one amino-terminal (N) domain of 108–110 amino acid residues homologous to Ig variable domains, followed by a differing number of Ig constant-like domains. CD66 mAbs react with members of the CEACAM family. Clearly characterized mAbs belonging to the CD66 cluster are described by their reactivity with each family member as indicated by a lower case letter after "CD66" as follows: CD66a mAb, CEACAM1, biliary glycoprotein; CD66b mAb, CEACAM8, CGM6; CD66c mAb, CEACAM6, NCA; CD66d mAb, CEACAM3, CGM1; and CD66e mAb, CEACAM5 or CEA [3]. CEACAM-1, -3, -6, and-8, but not CEACAM-5 (CEA), are expressed on human neutrophils. In humans, at least eight forms of CEACAM1, produced by differential splicing of the single CEACAM1 gene, have been identified [51-55]. In neutrophils, CEACAM1 and CEACAM3 exist as transmembrane proteins with cytoplasmic tails, while CEACAM8 and CEACAM6 are linked to the membrane via a glycosyl-phosphatidylinositol anchor.

CD66 mAbs have been reported to activate neutrophils [23,24,27,37,39-41]. By use of specific mAbs, each of the CEACAM family members expressed on neutrophils, CEACAM1, CEACAM8, CEACAM6, and CEACAM3 (recognized by CD66a, CD66b, CD66c, and CD66d mAbs, respectively) have been shown to be capable of activating neutrophils as determined by the physiologic response of adhesion to human umbilical vein endothelial cells (HUVECs) [37]. CD66 mAb binding to the neutrophil surface triggers a transient activation signal that requires extracellular calcium and regulates the adhesive activity of CD11/CD18 [37]. In the absence of extracellular calcium, this activation state decays and is no longer functional after 10 min.

The similarity in structure among the CEACAMs, and their ability to undergo homotypic and heterotypic interactions with other members of the family, led us to question the degree of interdependency of CEACAM signaling in neutrophils. To examine potential interactions among CEACAM members in transmitting signals in neutrophils, the effects of a set of well characterized CD66 mAbs on neutrophil adhesion to HUVECs was studied. The ability of combinations of CD66 mAbs, in the absence of calcium, to desensitize neutrophils to subsequent simulation by CD66 mAbs was examined. The data demonstrate significant functional independence of the four CEACAM molecules in signaling, but also suggest a unique role for CEACAM-1 in CEACAM signaling in neutrophils.

## Methods

### Cell preparation

Normal peripheral blood neutrophils were prepared by a modification of the method of Boyum as previously described [56], and were suspended at the indicated concentrations in Hanks' balanced salt solution (HBSS) with or without Ca^2+ ^(Gibco, Grand Island, NY), as indicated. Differential cell counts on Wright-stained cells routinely revealed greater than 95% neutrophils. Viability as assessed by trypan blue dye exclusion was greater than 98%.

### Antibodies and reagents

The CD45 mAb AHN-12 (IgG1) [57], the CD63 mAb AHN-16.1 (IgG1) [58], and the anti-HLA class I mAb W6/32 (IgG2a) [59] have been previously described. CD66 mAbs were obtained from the CD66 section of the Sixth International Workshop and Conference on Human Leukocyte Differentiation Antigens and included the following CD66 mAbs: B13.9 (IgG1) (CD66b), C11228.2C (IgG1) (CD66c), Bu-104 (IgG1) (CD66ae), and COL-1 (IgG2a) (CD66de) [3].

The PE-labeled CD11b mAb (Leu 15) was obtained from Becton Dickenson (Mountain View, CA). The source of mAbs was either hybridoma cell culture supernatants, purified antibody, or ascites fluid diluted in PBS containing 1 mg/ml BSA as indicated. All sera and ascites were heat inactivated at 56°C for 30 min and clarified by centrifugation at 13,000 × g at 4°C for 15 min before use. N-formyl-met-leu-phe (FMLP) and normal mouse serum (NMS) were purchased from Sigma Chemical Co. (St. Louis, MO).

### Fluorescence labeling of cells

Neutrophils were labeled with calcein AM (Molecular Probes, Eugene, OR) [60] by incubating 5 × 10^6^ cells/ml  with 50 ug of calcein AM for 30 min at 37°C in 18 ml of calcein labeling buffer [HBSS without Ca^2+ ^or Mg^2+ ^containing 0.02% BSA]. Cells were then washed twice with calcein labeling buffer at 23°C and resuspended in the desired media.

### Endothelial cell adhesion assay

**Figure 1:**
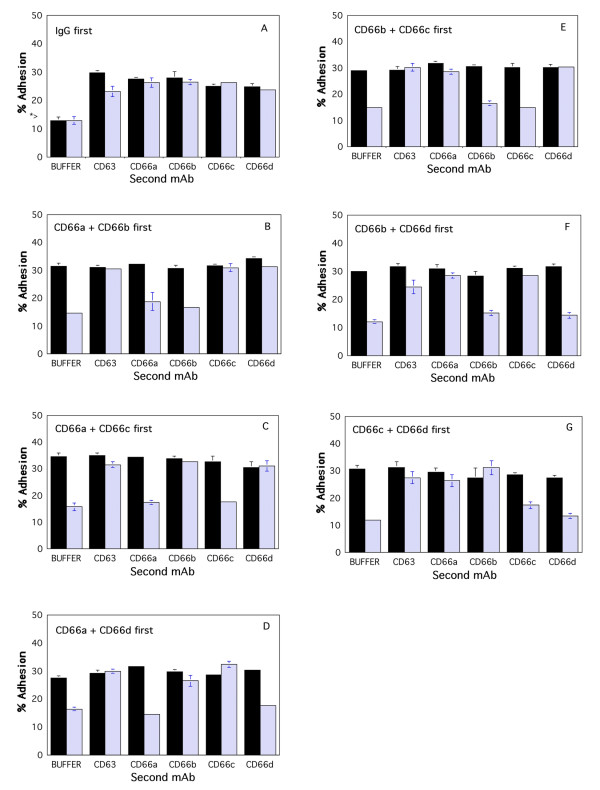
Cross desensitization with two CD66 mAbs to further stimulation of neutrophil adhesion to HUVECs. TNF-stimulated HUVECs were washed, and Ca^2+ ^free buffer containing IgG (panel A), the CD66ae mAb and CD66b mAb (panel B), the CD66ae mAb and CD66c mAb (panel C), the CD66ae mAb and CD66de mAb (panel D), the CD66b mAb and CD66c mAb (panel E), the CD66b mAb and CD66de mAb (panel F), or the CD66c mAb and CD66de mAb (panel G), were added (see Methods). Neutrophils in Ca^2+ ^free buffer were then added. After 15 sec (solid bars) or 15 min (hatched bars), the indicated next mAb or buffer, and Ca^2+ ^(1.8 mM final concentration) were added. After 30 min the wells were washed. The * > (Panel A) indicates the amount of adhesion observed when neutrophils were incubated in the wells for 30 min in the presence of buffer containing Ca^2+ ^with or without 10 ug/ml IgG (final concentration). The percent of neutrophils adherent to the monolayers are shown. Selective desensitization at 15 min was statistically significant (p < 0.05).

**Figure 2:**
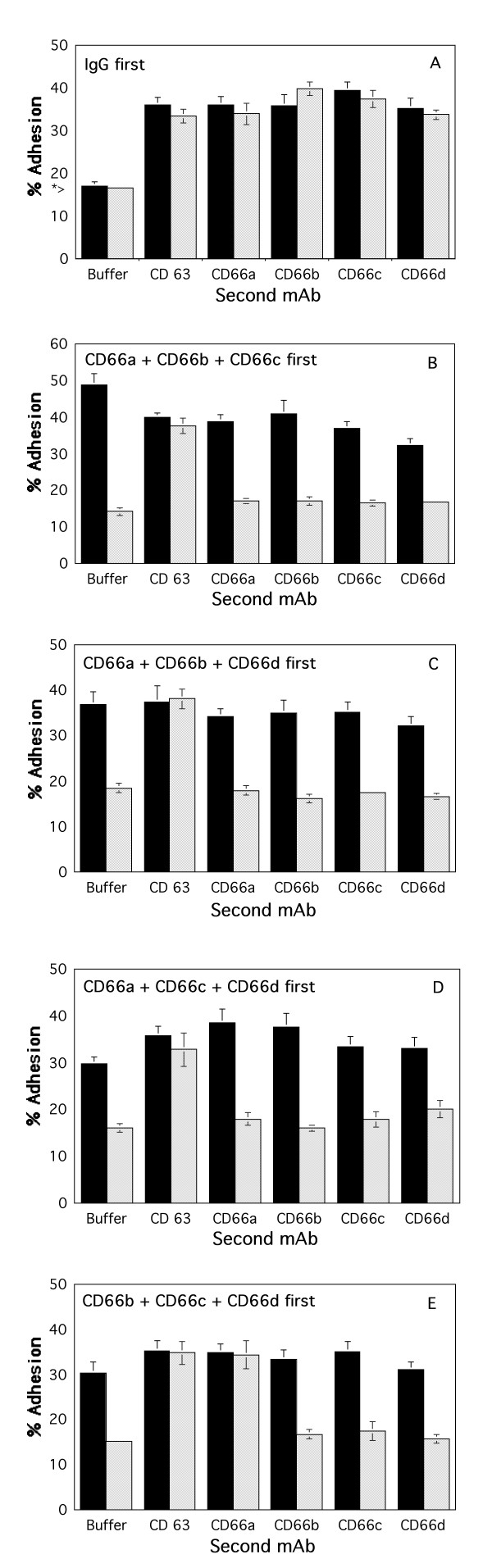
Cross desensitization with three CD66 mAbs to further stimulation of neutrophil adhesion to HUVECs. TNF-stimulated HUVECs were washed and Ca^2+ ^free buffer containing 10 ug/ml final concentration each of IgG (panel A), the CD66ae mAb, CD66b mAb, and CD66c mAb (panel B), the CD66ae mAb, CD66b mAb, and CD66de mAb (panel C), the CD66ae mAb, CD66c mAb, and CD66de mAb (panel D), or the CD66b mAb, CD66c mAb, and CD66de mAb (panel E), were added (see Methods). Neutrophils in Ca^2+ ^free buffer were then added. After 15 sec (solid bars) or 15 min (hatched bars), buffer containing 10 ug/ml final concentration of the indicated next mAb or buffer, and Ca^2+ ^(1.8 mM final concentration) were added. After 30 min the wells were washed. The * > (panel A) indicates the amount of adhesion observed when neutrophils were incubated for 30 min in the presence of buffer containing Ca^2+ ^with or without 10 ug/ml IgG (final concentration). The percent of neutrophils remaining adherent are shown. Selective desensitization at 15 min was statistically significant (p < 0.05).

Neutrophil adhesion to human umbilical vein endothelial cells (HUVECs) was performed as previously described [37]. Briefly, HUVECs (Clonetics Corp., San Diego, CA) were passaged 1:5 in T-25 flasks (Costar) no more than three times before plating in 96 well microtiter plates at 3000 cells/well. HUVECs were grown to confluence in 96 well microtiter plates in EGM media (Clonetics) and fed every 24 hours. Using the adhesion assay described below, no difference in resting and stimulated neutrophil adhesion was observed, and, as expected [37,61], no difference in surface expression of CD54 (ICAM-1) or CD62E (E selectin, ELAM-1) in resting or TNF stimulated cells was noted, using HUVECs passaged once compared with those passaged five times. In some experiments, the HUVECs were stimulated by culture for 4 hours at 37°C with 50 ng/ml TNFα (Cetus, Emeryville, CA). The wells were then washed four times with calcium free wash buffer (HBSS without Ca^2+ ^plus 4% HIFBS) and 25 ul of calcium free wash buffer containing the indicated antibody (10 ug/ml final concentration) was added to each well. One hundred ul of calcium free wash buffer containing 10^5 ^calcein-labeled neutrophils was added. After the indicated time, 25 ul of calcium-free wash buffer containing the indicated mAb (10 ug/ml final concentration) and 10.8 mM Ca^2+ ^was then added to yield a final physiologic calcium concentration (1.8 mM), and the plates were incubated at 37°C in 5% CO_2 _for 30 min. The wells were then aspirated and washed 4 times with endo wash buffer (HBSS plus 4% HIFBS), and the fluorescence was quantitated with a Millipore fluorescence plate reader using an excitation wavelength of 485 nm and an emission wavelength of 530 nm. For each condition, quadruplicate wells were tested and values are reported as the mean +/- SD. Each experiment was performed at least four times using different HUVEC subcultures. The data in Figures 1 and 2 are shown as the percent of added neutrophils remaining adherent to the monolayers, and represent the means +/- SD of 4 separate determinations. While the SD is shown in each figure, in some panels it is sufficiently small that it is not possible to see on the scale shown.

### Statistical analyses

Effects of mAbs on neutrophil adhesion to HUVECs was analyzed by the Mann Whitney U test when appropriate.

## Results

### Effects of CD66 mAbs on neutrophil adhesion to endothelial cells

Because CEACAM-1, -3, -6, and -8 are highly homologous structurally, and can undergo a number of different homotypic and heterotypic adhesion reactions among themselves [2,26,62-72], it is possible that they might interact on the neutrophil surface. To better characterize possible interactions among the CEACAMs in signaling on human neutrophils, we utilized calcium-dependent desensitization by CD66 mAbs to examine individual CEACAM-mediated signaling. As expected, when neutrophils were incubated for 30 min with HUVECs and 10^-7 ^M FMLP in the presence of normal mouse IgG (IgG) or mAb, and washed as described in the Methods, each of the CD66ae, CD66b, CD66c, CD66de, and the control CD63 mAbs augmented neutrophil adhesion approximately two-fold compared with IgG or media [not shown and [37,58]]. In contrast, neither the CD45 mAb nor the anti-HLA class I mAb altered neutrophil adhesion (not shown).

### Cross desensitization to pairs of CD66a, CD66b, CD66c, and CD66d mAbs

Desensitization of neutrophils to further stimulation by mAbs directed to specific CEACAM family members by exposure of the neutrophils to the mAbs in the absence of calcium was used to examine the independence of signaling mechanisms triggered by each CD66 mAb. Although these CD66 mAbs stimulated neutrophil adhesion to resting HUVEC [37], for the experiments reported here, TNF-treated HUVECs were used because these conditions yielded a stronger signal in the assay. HUVECs were stimulated for 4 hours with 50 ng/ml TNFα, washed, and neutrophils were added with desensitizing mAbs, incubated in the absence of calcium, washed, and stimulated with other mAbs and cell adhesion quantitated as described in the Methods. First, IgG was added to the microtiter wells containing the TNF stimulated HUVECs in the absence of Ca^2+ ^(Fig 1, panel A). As expected [37,58], when neutrophils were added to the wells in the absence of Ca^2+ ^and allowed to incubate for 15 sec before Ca^2+ ^was added (solid bars) stimulated neutrophil adhesion was observed when aliquots of CD66ae mAb, CD66b mAb, CD66c, CD66de, or CD63 mAbs were added, but not when buffer was added. Since the CD66e antigen is not expressed in neutrophils, the available CD66ae and CD66de mAbs can be used effectively as CD66a and CD66d mAbs, respectively, in this cell system. When neutrophils were added to the wells in the absence of Ca^2+ ^and allowed to incubate for 15 min before Ca^2+ ^was added (hatched bars), stimulated neutrophil adhesion to the HUVECs following the addition of aliquots of CD66ae, CD66b, CD66c, CD66de, and CD63 mAbs, but not buffer was also observed.

Next, the CD66ae and CD66b mAbs were added to the microtiter wells containing the TNF stimulated HUVECs in the absence of Ca^2+ ^(Fig 1, panel B). As expected, when neutrophils were added to the wells in the absence of Ca^2+ ^and allowed to incubate for 15 sec before Ca^2+ ^was added (solid bars) stimulated neutrophil adhesion was observed when aliquots of buffer, CD66ae mAb, or CD66b mAb, were added. Adhesion was also observed when aliquots of CD66c mAb, CD66de mAb, or CD63 mAb were added. When neutrophils were added to the wells in the absence of Ca^2+ ^and allowed to incubate for 15 min before Ca^2+ ^was added (hatched bars), there was a marked decrease in neutrophil adhesion to the HUVECs following the addition of aliquots of buffer, CD66ae mAb, or CD66b mAb. In contrast, the cells were still responsive to stimulation by CD66c, CD66de, and CD63 mAbs as evidenced by an increase in adhesion.

Similarly, desensitization of neutrophils to stimulation by the CD66ae and CD66c mAbs selectively desensitized the cells to further stimulation by the CD66ae mAb and the CD66c mAb, but not by CD66b, CD66de, or CD63 mAbs (Fig 1, panel C). Finally, desensitization to the CD66ae and CD66de mAbs left the cells unresponsive to CD66ae and CD66de mAbs, but they remained responsive to CD66b, CD66c, and CD63 mAbs (Fig 1, panel D).

When cells were desensitized to CD66b and CD66c mAbs, the cells were unresponsive to CD66b and CD66c mAbs, but were still responsive to stimulation by CD66ae, CD66de, and CD63 mAbs as evidenced by an increase in adhesion (Fig 1, panel E). Similarly, desensitization of neutrophils to stimulation by the CD66b and CD66de mAbs selectively desensitized the cells to further stimulation by the CD66b and CD66de mAbs, but not by CD66ae, CD66c, or CD63 mAbs (Fig 1, panel F). Similar selectivity of this desensitization was observed when cells were desensitized with the CD66c mAb and the CD66de mAb, in that the cells were desensitized to CD66c and CD66de mAbs, but not to CD66ae, CD66b, or CD63 mAbs (Fig 1, panel G).

### Cross desensitization to combinations of three CD66 mAbs

Desensitization with various combinations of three CD66 mAbs was next examined. First, IgG was added to the microtiter wells containing the TNF stimulated HUVECs in the absence of Ca^2+ ^(Fig 2, panel A). As expected, when neutrophils were added to the wells in the absence of Ca^2+ ^and allowed to incubate for 15 sec (solid bars) or 15 min (hatched bars) before Ca^2+ ^was added, stimulated neutrophil adhesion was similar to that observed in Figure 1, panel A. Next, the CD66ae, CD66b, and CD66c mAbs were added to the microtiter wells containing the TNF stimulated HUVECs in the absence of Ca^2+ ^(Fig 2, panel B). As expected, when neutrophils were added to the wells in the absence of Ca^2+ ^and allowed to incubate for 15 sec before Ca^2+ ^was added (solid bars), stimulated neutrophil adhesion was observed when aliquots of buffer, CD66ae mAb, CD66b mAb, CD66c mAb, CD66de mAb, or CD63 mAb were added. When neutrophils were added to the wells in the absence of Ca^2+ ^and allowed to incubate for 15 min before Ca^2+ ^was added (hatched bars), there was a marked decrease in neutrophil adhesion to the HUVECs following the addition of aliquots of buffer, CD66ae mAb, CD66b mAb, or CD66c mAb. In addition, the cells were no longer responsive to stimulation by the CD66de mAb. In contrast, the cells were still responsive to stimulation by CD63 mAbs as evidenced by an increase in adhesion. Thus, cells were desensitized to CD66de mAb stimulation with a combination of mAbs that does not bind the CD66d antigen. Similarly, desensitization of neutrophils to stimulation by the CD66ae, CD66b, and CD66de mAbs desensitized the cells to further stimulation by the CD66c mAb, as well as CD66ae, CD66b, and CD66de mAbs, but not by CD63 mAbs (Fig 2, panel C). Similar selectivity of this desensitization was observed when cells were desensitized with the CD66ae, CD66c, and CD66de mAbs, in that the cells were desensitized to CD66ae, CD66b, CD66c, and CD66de mAbs, but not to CD63 mAbs (Fig 2, panel D). In contrast, desensitization to the CD66b, CD66c, and CD66de mAbs left the cells unresponsive to CD66b, CD66c, and CD66de mAbs, but they remained responsive to both CD66ae and CD63 mAbs (Fig 2, panel E).

## Discussion

While it has been shown that CEACAM-1, -8, -6, and -3 can each independently transduce signals in neutrophils resulting in activation of CD11/CD18, and an increase in neutrophil adhesion to endothelial cells [37], potential interactions among these molecules in neutrophil activation are not well defined. Experiments in which CD66 mAbs were allowed to bind to the neutrophils for various lengths of time in the absence of calcium before calcium repletion, suggested that the binding of CD66 mAbs to the neutrophil surface results in a transient activation state during which time a signal can be transmitted to CD11/CD18 if extracellular calcium is present. In the absence of extracellular calcium, this activation state decayed significantly within 1 min, and is no longer functional after 10 min, i.e. the cell is desensitized to stimulation by that mAb [37]. This observation allowed the current study to be performed.

This study demonstrates that desensitization of neutrophils to stimulation by any two neutrophil CEACAMs allows the cell to respond to stimulation by the other two neutrophil CEACAMs. However, neutrophils desensitized to CEACAM-1 and any other two neutrophil CEACAMs, are unresponsive to the remaining neutrophil CEACAM, while retaining responsiveness to the unrelated membrane protein CD63. In contrast, neutrophils desensitized to CEACAM-8, -6, and -3, were still responsive to both CEACAM-1 and CD63. Thus, CEACAM-1 appears to have a unique role in CEACAM signaling in neutrophils.

We feel the observed results are due to mAbs binding their specific antigens on the neutrophil surface. There are potential alternative explanations for the results observed in this study. CEACAM1 can be expressed on HUVECs. Therefore, in earlier studies, a series of experiments addressed the possibility that the observed results could be due to CD66 mAb binding the HUVECs [37]. Preincubation of HUVECs with mAb under various conditions, followed by washing, indicated that the effects of CD66 mAbs were due to mAbs binding to the neutrophils and not the HUVECs [37].

Furthermore, it was also possible that the Fc fragments of these mAbs could alter signaling. The CD66 mAbs used here could also induce a conformational change in a CEACAM, or possibly cluster surface CEACAMs. These possibilities were addressed in an earlier report in which F(ab')_2 _fragments of the CD66ae, CD66be, and CD66c mAbs were found to stimulate neutrophil adhesion to HUVECs in this assay, as did the intact IgGs [37]. In contrast, Fab fragments of the CD66ae mAb had little effect on neutrophil adhesion in this assay, suggesting that cross-linking or clustering of CEACAMs could play a role in the observed effects [37].

The molecular explanation for these observations is unclear. CD66b and CD66c mAbs triggered an activation signal, despite the fact that they bind GPI-linked surface proteins, as has been previously reported [37]. MAb binding to other GPI-liniked proteins can also transduce signals [27]. While the details of the "activation signal" transmitted by CEACAMs are not known, the finding of tyrosine kinase activity, including lyn and hck, associated with CEACAM-1, CEACAM-6, and CEACAM-8, and src with CEACAM-1, suggests that these kinase activities may be involved in signal transduction via CEACAM family members [73,74]. CEACAM1 is also associated with protein tyrosine phosphatase activity [75]. CEACAM1 in neutrophils also undergoes transient changes in phosphorylation following stimulation with chemotactic agents, suggesting that phosphorylation may be involved in regulating CEACAM-1 function as well [73,74]. CEACAM3 is tyrosine phosphorylated upon binding gonococci expressing CEACAM ligand Opa protein variants [76]. Tyrosine kinase activity in neutrophils has also been reported to be associated with CD63, the control signaling molecule used in this study [58], while serine kinase activity has been reported to associate with CD63 in melanoma cells [77].

Although mAbs to both CEACAM1 and CEACAM3 triggered neutrophil activation in this study, the cytoplasmic domain of CEACAM1 has an ITIM motif, while that of CEACAM3 contains an ITAM sequence. In a transfected HeLa epithelial cell model, uptake of gonococci mediated by CEACAM1 and CEACAM3 differed with regard to their sensitivity to tyrosine kinase inhibitors [78]. Other studies have also found differences in the mechanism of CEACAM3 and CEACAM6 mediated uptake; the former being dependent on tyrosine kinase activity and the latter requiring the integritiy of cholesterol-rich membrane microdomains [79,80].

**Figure 3:**
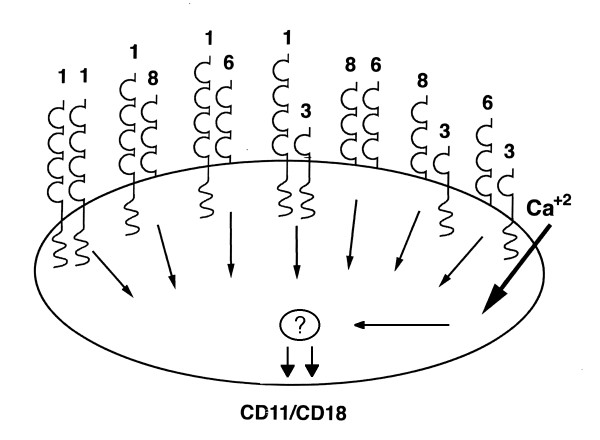
Model of potential CEACAM dimers in signaling complexes on neutrophils. A possible model of CEACAM signaling complexes on neutrophils that is compatible with observed desensitization data is shown. In this model, CEACAMs can exist on the neutrophil surface as heterodimers or as CEACAM-1 homodimers. Signaling would require an active dimer. For example, desensitization of CEACAM-1 would allow signaling by CEACAM-8/6; 8/3; or 6/3 dimers, while desensitization of CEACAM-1 and any other two CEACAMs would leave no active dimers. In contrast, desensitization of CEACAMs-8, 6, and 3 would leave active CEACAM-1 homodimers. The existence of potential unidentified cooperative signaling molecules is denoted by the "?"

The data are consistent with the existence of signaling complexes containing more than one CEACAM on the neutrophil surface. CEACAMs have been shown to undergo homotypic and heterotypic adhesion [55,62,65-67,70-72,81-83]. CEACAM8 exhibits heterotypic adhesion with CEACAM6, while CEACAM-1, -6, and -5 exhibit both homotypic and heterotypic adhesion. For example, a model in which CEACAMs exist as heterodimers containing two different CEACAMs or CEACAM-1-CEACAM-1 homodimers in a signaling complex, in which an active CEACAM dimer is required for signal transmission, could explain the current observations (Fig 3). For example, in this model, desensitization of CEACAM-1 would allow signaling by CEACAM-8/6; 8/3; or 6/3 dimers, while desensitization of CEACAM-1 and any other two CEACAMs would leave no active dimers. In contrast, desensitization of CEACAMs-8, 6, and 3 would leave active CEACAM-1 homodimers. Association of CEACAMs into larger complexes containing more than just two CEACAMs is also possible. Data have been reported showing that CEACAM-1 can form dimers in solution and on an epithelial cell surface [84]. Dr. Singer and colleagues have provided evidence that complex formation among CEACAMs in neutrophils is possible [35,85]. Despite having tried a number of experimental approaches, including immunoprecipitation, immunoblotting, and surface labeling with ^125^I and biotin, we have not been able to detect the existence of such complexes in neutrophils (data not shown). Given the convergence of signaling by the different CEACAMs with different cytoplasmic domains, it is possible that another molecule may act as an intermediary in CEACAM signaling.

The role(s) of CEACAMs in neutrophil function are complex. However, ligation of CEACAM-1, -8, -6, and -3 by CD66a, CD66b, CD66c, and CD66d mAbs, respectively, transduce signals in neutrophils resulting in activation of CD11/CD18, and an increase in neutrophil adhesion to endothelial cells, one of the critical first steps of inflammation [37]. In addition, several other reports have also suggested that CEACAMs are capable of regulating the function of CD11/CD18 [24,39,40], and induce an increase in intracytoplasmic calcium and an oxidative burst in neutrophils [27]. CEACAM1 also regulates neutrophil apoptosis, thus possibly influencing the resolution of inflammation [34]. Finally, studies have shown that certain bacteria bind to some CEACAM family members on neutrophils, and this interaction may also result in signal transduction resulting in modification of neutrophil activity [6,8,22,86-96]. Thus, CEACAMs appear to be involved in neutrophil adhesion by transmitting some form of activation signal that regulates the activity of other adhesion molecules, as well as possibly by homotypic or heterotypic adhesion. CEACAMs-1, -8, and -6, are upregulated to the neutrophil surface from intracellular stores following stimulation [97-99].

The current observations may also be relevant to other cells expressing CEACAMs. CEACAM1 and CEACAM6 have been reported to present selectin ligands to CD62E (ELAM-1, E-selectin) on endothelial cells [23], and appear to be involved in angiogenesis [9,16,28,100]. A role for a soluble form of CEACAM1 in angiogenesis has also been demonstrated [100]. CEACAM1 also appears to play a critical role in tumor lymphangiogenesis [15], and can regulate cell migration via interaction with filamin A [17]. CEACAM1 associates with the beta 3-integrin, and this association is dependent on the phosphorylation of Tyr-488 in the cytoplasmic domain of CEACAM1; this complex may play a role in cell invasion [101]. During cell-matrix adhesion of endothelial cells, CEACAM1 associates with talin, a regulator of integrin function [28]. CEACAMs serve as a receptor for murine hepatitis virus [102-106], and as a human receptor for *Neisseria meningiditis *and *Neisseria gonorrhea *[8,22,86-91,94,95]. CEACAMs can also transmit signals regulating proliferation of epithelial cells and lymphocytes [2,6-8,13,14,22,35,36,107,108]. Thus, interactions among CEACAMs in signaling may occur in various cell systems.

## Competing interests

The authors declare that they have no competing interests.

## Authors' contributions

KMS participated in study design, data analysis, and helped draft the manuscript.

APNS participated in study design, data analysis, and helped draft the manuscript.

All authors read and approved the manuscript.
